# Quality of Life and Patient Satisfaction 3 Months and 3 years After Laparoscopic Nissen's Fundoplication

**DOI:** 10.4103/1319-3767.37799

**Published:** 2008-01

**Authors:** Emad Hamdy, Ahmed Abd El-Raouf, Mohamed El-Hemaly, Tarek Salah, Ehab El-Hanafy, Mohamed Mostafa, Nabil GadEl Hak

**Affiliations:** Gastroenterology Surgical Center, Mansoura University, Mansoura, Egypt

**Keywords:** Laparoscopic Nissen's fundoplication, quality of life

## Abstract

**Background/Aim:**

This study is based on studying the quality of life and degree of satisfaction among gastroesophageal reflux disease (GERD) patients after Laparoscopic Nissen's fundoplication (LNF) operations.

**Summary Background Data:**

A GERD patient is most interested in symptom relief, whereas his surgeon will also be interested in the improvement of anatomical and functional investigations. Materials and Methods: One hundred patients with symptoms of GERD, adequate motility study and positive 24-hour pH studies underwent LNF in El-Mansoura Gastroenterology Center between 2002 and 2004. All patients completed the Gastroesophageal Reflux Disease-Health Related Quality Of Life (GERD-HRQL) questionnaire both pre- and postoperatively (early within 3 months after operation and late after 3 years). Furthermore, all patients were given a form with 4 grades (excellent, good, fair and poor) and they were requested to freely assess both the early and late clinical outcomes.

**Results:**

There was statistically significant improvement in all the items of the GERD-HRQL questionnaire, both early and late (*P* < 0.001). With regard to patient satisfaction; only 58 cases showed excellent clinical satisfaction early postoperatively, while 29, 8 and 5 patients showed good, fair and poor responses, respectively. These figures improved on late followup (*P* < 0.05), i.e., 76 excellent, 16 good and 8 fair results.

**Conclusions:**

LNF improves all the items of quality of life among GERD patients significantly (*P* < 0.001). Patient satisfaction after LNF improves with time; 58 cases showed early postoperative excellent clinical satisfaction as compared to 76 late cases (*P* < 0.05). However, LNF may not be the standard management of reflux symptoms, particularly from some patients' perspective.

Most surgical operations are designed to correct or change anatomical arrangements, while antireflux operation is designed to correct a physiological state. Therefore, the evaluation of the efficacy of antireflux surgery may focus on symptoms, signs of damage (endoscopy), physiological abnormalities (manometry and pH studies), global assessments or some combination of the above.[1]

Health-related quality of life is becoming increasingly important as an outcome measure of treatment since neither the questioning of symptoms alone (such as heartburn, regurgitation or dysphagia) nor the assessment of objective findings such as endoscopic evaluation, esophageal sphincter manometry or pH monitoring seem to adequately reflect the subjective well-being of the patients. The expense and inconvenience of investigations also have to be considered.[2]

Symptom severity can be measured with the Gastroesophageal Reflux Disease-Health Related Quality Of Life (GERD-HRQL) questionnaire. This questionnaire is based on descriptive anchors and has been previously shown to be a valid, reliable, responsive and practical measure of GERD symptoms.[3]

Despite the majority of patients being well satisfied after surgery, up to 10% of patients would not recommend the procedure to others. The reasons for dissatisfaction include prolonged dysphagia, gas bloat symptoms or recurrent reflux. Postoperative dysphagia is generally short-lived and few patients have long-term dysphagia, as described elsewhere.[4]

Some patients, however, do not have an identifiable cause for persistent postoperative symptoms. Certain characteristics of these individuals have been identified, and they include a relatively poor indication for surgery (mildly abnormal pH study without erosive esophagitis), coexisting psychiatric diagnoses such as depression or anxiety and chronic pain syndromes such as fibromyalgia. In both the community and academic settings, these patients should not be offered laparoscopic fundoplication because of a high likelihood of persistent postoperative symptoms.[5]

## MATERIALS AND METHODS

This prospective study included 100 patients with GERD who were operated upon by LNF in El-Mansoura Gastroenterology Surgical Center in the period between January 2000 and March 2002 (out of 800 GERD patients treated during this period – either medical or surgical cases). All the operations were performed by the same surgeon. These included 61 (61%) males and 39 (39%) females. The mean age was 36.1 ± 9.5 years (range: 18–64 years).

### Criteria of selection

Most of the patients included in this study had typical reflux symptoms, documented reflux by upper endoscopy and barium study, low lower esophageal sphincter pressure (LESP) and pathological acid reflux by 24-hour pH study.

### Criteria of exclusion

Patients with a history of previous gastric or esophageal surgeries, complicated refluxes (e.g., ulcer, stricture or short esophagus) or manometrically diagnosed concurrent systemic diseases (e.g., scleroderma, achalasia or other primary esophageal motility disorders) were not considered in the study as we prefer antireflux medical treatment for such cases.

### Preoperative assessment

All patients were subjected to thorough clinical evaluation, upper endoscopy, barium study, esophageal manometry and 24-hour pH-metry. Forty patients underwent 24-hour bilitec study. All preoperative data was compared with the postoperative scores.

### Operative technique

All cases underwent LNF. After satisfactory hiatal dissection, hiatal repair was performed, leaving a space that nearly equals the esophageal diameter. Short gastric vessels were divided in all cases creating a short floppy wrap that had a length of 2 cm without the use of a dilator in the esophagus.

### Evaluation of quality of life

All patients completed the GERD-HRQL questionnaire both pre- and postoperatively. Each item is scored from 0 (best score) to 5 (worst score). The total GERD-HRQL score is simply the sum of all the 10 items (best possible score: 0, worst possible score: 50).

### The GERD-HRQL scale[6]

Scale

0 = No symptoms.
1 = Symptoms noticeable, but not bothersome.
2 = Symptoms noticeable and bothersome, but not everyday.
3 = Symptoms bothersome everyday.
4 = Symptoms that affect daily activities.
5 = Symptoms are incapacitating (unable to perform daily activities).

Questions

1. How bad is your heartburn?1 2 3 4 52. Heartburn when lying down?1 2 3 4 53. Heartburn when standing up?1 2 3 4 54. Heartburn after meals?1 2 3 4 55. Does heartburn change your diet?1 2 3 4 56. Does heartburn wake you from sleep?1 2 3 4 57. Do you have difficulty swallowing?1 2 3 4 58. Do you have pain with swallowing?1 2 3 4 59. Do you have gassy or bloating feelings?1 2 3 4 510. If you take medication, does it affect your daily life?1 2 3 4 5

### Postoperative assessment

All patients were asked to pay followup visits at least twice: the first followup visit was within the first 3 months after surgery (early followup) and the second one was after 3 years (late followup). During followup, each patient was assessed in the manner similar to that of preoperative assessment, i.e., clinical evaluation, endoscopic examination, radiological study, esophageal manometry and 24-hour pH-metry. All the patients were clinically assessed both early and late. Ninety and then 80 cases underwent anatomical and physiological assessment early and late, respectively.

### Patient satisfaction

Furthermore, all the patients were given a form showing 4 grades (excellent, good, fair and poor) and they had to freely evaluate the clinical outcome both early (< 3 months) and late (> 3 years) in the postoperative period [Table 1].[7]

**Table 1:** Grading of overall patient satisfaction

Grade	Description
Excellent	Completely recovered
Good	Major improvement with minor problems
Fair	Major improvement, but still some significant symptoms or side effects
Poor	Minor or no improvement or even worsening

Postoperative assessment and patient satisfaction data were collected by an independent team that did not take part in the operative procedures.

### Postoperative care

The majority of the patients started oral intake on the second postoperative day with a mean of 2.1 ± 0.3 days (range: 2–4 days). All patients were advised to have liquid or semisolid food for the first postoperative month. The mean duration of hospital stay was 3.3 ± 1.0 days (range: 2–6 days). Only 3 cases who developed severe distension stayed in the hospital for 6 days with a nasogastric tube and intravenous fluids for 4 days until the distension subsided and the oral intake was started.

### Statistical analysis

All values are expressed as means. Paired values were compared with student's t-test. A *P*-value less than 0.05 was considered to be statistically significant.

## RESULTS

### Quality of life

Table 2 presents the median preoperative, early and late postoperative median scores for each item of the GERD-HRQL. There was statistically significant improvement in items 1 through 6 and 10 both early and late (*P* < 0.001). Further, the median of the total score improved from 33 preoperatively to 3 early and 0 late (*P* < 0.001). When evaluating postoperative outcomes, and comparing early results with late ones, there were improvements noted in dysphagia (item 7), odynophagia (item 8) and bloating (item 9).

**Table 2:** Quality of life

Data	Preoperative Median score	Early postoperative Median score	Late postoperative Median score	*P*-value	
Item 1	5 (1–5)	0 (0–4)	0 (0–4)	*P*_1_ < 0.001	*P*_2_ < 0.001
Item 2	5 (1–5)	0 (0–4)	0 (0–4)		
Item 3	4 (0–5)	0 (0–3)	0 (0–3)		
Item 4	5 (0–5)	0 (0–3)	0 (0–3)		
Item 5	4 (0–5)	0 (0–3)	0 (0–3)		
Item 6	4 (0–5)	0 (0–3)	0 (0–3)		
Item 7	1 (0–4)	2 (0–4)	1 (0–3)		
Item 8	1 (0–5)	2 (0–4)	1 (0–3)		
Item 9	1 (1–5)	2 (0–4)	1 (0–3)		
Item 10	4 (1–5)	0 (0–3)	0 (0–3)		
Total score	33 (14–40)	3 (0–27)	0 (0–28)		
Total	100	100	100		

P_1:_ P-value between preoperative and early postoperative results. P_2:_ P-value between preoperative and late postoperative results.

### Patient satisfaction

Fifty-eight cases showed excellent clinical satisfaction in the early postoperative period, while 29 showed good responses (7: recurrent mild to moderate heartburn, 19: mild to moderate dysphagia and distension and 3: persistent, but decreased level of atypical symptoms), 8 showed a fair response (3 moderate dysphagia and distension and 5 persistent atypical symptoms) and 5 indicated a poor response (cases with tight wrap who needed dilatation). These figures improved on late followup (*P* < 0.05): 76 excellent, 16 good (9: recurrent mild to moderate heartburn, 6: mild to moderate dysphagia and distension and 1: persistent but decreased level of atypical symptoms) and 8 fair (2 recurrent severe heartburn, one significant dysphagia and distension and 5 persistent atypical symptoms) [Table 3]. It should be noted that the incidence of postoperative dissatisfaction was common among the cases that had preoperative atypical symptoms: 8 (44.45%) early and 6 (33.33%) late out of 18 cases (*P* < 0.05).

**Table 3:** Patient satisfaction

Data	Early postoperative No (%)	Late postoperative No (%)	*P*-value
Excellent	58 (58)	76 (76)	0.001
Good	29 (29)	16 (16)	
Fair	8 (8)	8 (8)	
Poor	5 (5)	0 (0)	
Total	100	100	

### Redo operation

Redo operation was performed for two cases: the one with recurrent severe heartburn due to wrap disruption (2 years postoperative) and one with severe significant dysphagia due to tight wrap (3 weeks postoperative).

## DISCUSSION

### Quality of life following surgery

If one asks a patient and an antireflux surgeon how to evaluate an antireflux operation, the answers may well differ. The patient is most interested in the relief of the symptoms, whereas the surgeon will also be interested not only in the relief of symptoms, but in the improvement of anatomical and functional investigations. A patient who suffers from severe heartburn does not care if the postoperative LES pressures will rise.[1]

Conventionally, GERD symptoms have been measured qualitatively as “mild, moderate, severe, etc.,” as demonstrated by a recent study. The difficulty with this method is the great variation in the interpretation of the meaning of these words to individual patients and physicians. In other words, what is “mild” to one person may be “severe” to another. This is why the use of descriptive anchors in an ordinal scale is so important. The GERD-HRQL was developed in this manner and has been found to be a reliable, valid and most importantly a practical and responsive questionnaire.[8]

In our series, we used GERD-HRQL scale on the basis of a 10-point questionnaire. There was statistically significant improvement in items 1 through 6 and 10 (concerned with the severity of heartburn and medication requirement), both early and late (*P* < 0.001). When evaluating postoperative outcomes, and comparing early results with late ones, there were improvements noted in dysphagia (item 7), odynophagia (item 8) and bloating (item 9) on comparing the early results with the late ones.

### Patient satisfaction

What should a patient expect from antireflux surgery? The expectation could be summarized as follows: to lose the symptoms of reflux disease and not to acquire troublesome new side effects of the antireflux surgery. The quality of life is to improve to the extent that the disappearance of reflux symptoms is not replaced by new symptoms such as dysphagia. In addition to the disappearance of symptoms, there should be a return to normal dietary habits.[1]

In a large series involving 157 patients that had LNF with a long mean follow-up period (49 months) Tucker *et al*. showed that 73.1% patients were completely satisfied, 22.8% somewhat satisfied and 5.3% were unsatisfied with surgery. Overall satisfaction with surgery was 94.7% and 66.2% would recommend the surgery to the others.[5]

Among our cases, 58 cases showed excellent clinical satisfaction early postoperative, while 29 showed good response (7 recurrent mild to moderate heartburn, 19 mild to moderate dysphagia to solid food only and distension and 3 persistent but decreased level of atypical symptoms), 8 showed fair response (3 moderate dysphagia to solid food only and distension and 5 persistent atypical symptoms) and 5 indicated poor response (cases with tight wrap who developed significant dysphagia to both solids and liquids). It should be noted that all the cases with early dysphagia showed high LES pressure on manometric study, and all of them responded to conservative measures (by reassuring that this dysphagia is due to postoperative edema) except for the 5 cases with poor results that needed endoscopic balloon dilatation. Similarly, all cases with abdominal distension responded to conservative measures that included refraining from swallowing air and chewing food well, besides advising them to use antiflatulant and antispasmodics.

All the abovementioned figures improved on late followup (*P* < 0.05): 76 excellent, 16 good (9 recurrent mild to moderate heartburn, 6 mild to moderate dysphagia and distension and one persistent but decreased level of atypical symptoms) and 8 fair (2 recurrent severe heartburn, one significant dysphagia and distension and 5 persistent atypical symptoms). Redo operation was performed for two cases; one with recurrent severe heartburn due to wrap disruption (2 years postoperative) and one with severe significant dysphagia due to tight wrap (3weeks postoperative).

## CONCLUSIONS

LNF improves all the items of quality of life among GERD patients significantly. In addition, when specifically evaluating postoperative outcomes, and comparing early results with late ones, there were improvements noted in dysphagia, odynophagia and bloating.

Patient satisfaction after LNF improves over time. Our study shows that LNF may not be the standard management of reflux-related symptoms for every patient, specifically when viewed from a patient's perspective.
